# Personality correlates of dispositional forgiveness: a direct comparison of interpersonal and self-forgiveness using common transgression scenarios

**DOI:** 10.3389/fpsyg.2023.1218663

**Published:** 2023-11-01

**Authors:** Lucas E. Hampton, Nicholas P. Carruth, John H. Lurquin, Akira Miyake

**Affiliations:** ^1^Department of Psychology and Neuroscience, University of Colorado Boulder, Boulder, CO, United States; ^2^Department of Psychology, DePaul University, Chicago, IL, United States

**Keywords:** self-forgiveness, interpersonal forgiveness, Big Five, agreeableness, neuroticism, conscientiousness, explanatory style

## Abstract

Although the personality correlates of dispositional interpersonal forgiveness (forgiveness of others) have been well characterized, those of dispositional self-forgiveness are less well understood. Moreover, when the personality correlates are examined for both types of forgiveness, the comparison has been based on participants’ self-report ratings on questionnaires. The current study sought to address these gaps in the literature by adopting a scenario-based approach, which has been used less frequently, especially in self-forgiveness research. A total of 160 participants read six fictional scenarios, each describing a severe transgression, from the perspective of the transgressor (self-forgiveness, *n* = 78) or the victim (interpersonal forgiveness, *n* = 82) of the transgression, and then responded to several items assessing different facets of forgiveness (avoidance, revenge, and benevolence). Participants’ personality (Big Five) and explanatory style were also assessed. Consistent with prior literature, agreeableness and neuroticism generally predicted different facets of interpersonal forgiveness. These two personality traits also predicted facets of self-forgiveness, but, additionally, conscientiousness and one’s tendency to internalize failure (the personal component of explanatory style) uniquely predicted self-forgiveness, especially avoidance motivations. These results point to both similarities and differences in the personality correlates of interpersonal and self-forgiveness. As a secondary, more exploratory aim, the current study compared the results from our scenario-based assessment of forgiveness to those based on a commonly used questionnaire, the Other and Self subscales of the Heartland Forgiveness Scale (HFS). As expected, the Other subscale of the HFS were associated with levels of interpersonal forgiveness assessed with our transgression scenarios, but, surprisingly, the HFS Self subscale was more strongly related to interpersonal than self-forgivess assessed with scenarios. Moreover, the Self subscale was not associated with levels of self-forgiveness assessed with transgression scenarios, except for avoidance motivations. These results suggest that scenario-based and questionnaire-based methods may capture different facts of forgiveness and cannot be used interchangeably, especially for the assessment of self-forgiveness. More generally, the current study illustrates the importance of conducting direct within-study comparisons of interpersonal and self-forgiveness as well as of different assessment methods to better understand the similarities and differences between the two types of forgiveness.

## 1. Introduction

Imagine that a good friend of yours asks you to look after his beloved dog while he is on vacation. Before he leaves, your friend tells you that the dog likes to sneak out of the house and play outside. On the day of his return, you carelessly left the house door slightly ajar. The dog goes outside, gets hit by a car on the street, and dies. How would you feel? Would you be able to forgive yourself, knowing that your carelessness led to the dog’s death?

Now, imagine that the roles are reversed: It is your close friend, not you, who carelessly left the house door ajar and contributed to the death of your beloved dog. How would you feel? Would you be able to forgive your friend?

Although rare, such tragic and disastrous events can happen, necessitating either the forgiveness of the self (hereafter, *self-forgiveness*) or the forgiveness of others (hereafter, *interpersonal forgiveness*). It has been well known that both types of forgiveness are important in people’s everyday lives (for recent overviews of the forgiveness literature, see Worthington and Wade, 2020; Pettigrove and Enright, 2023). In particular, as one can easily imagine from the above “pet death” scenario, unexpectedly becoming the unintentional transgressor or victim of a severe transgression is likely to take its toll on their psychological well-being. Indeed, a wealth of evidence has documented substantial associations between forgiveness and mental health (Griffin et al., 2015; Webb and Toussaint, 2020), both in interpersonal forgiveness research (e.g., Barcaccia et al., 2020; Gismero-González et al., 2020; see Gao et al., 2022, for a meta-analysis) and in self-forgiveness research (e.g., Peterson et al., 2017; Carpenter et al., 2020; see Davis et al., 2015, for a meta-analysis).

Despite such importance of both types of forgiveness in people’s psychological well-being and mental health and in various other aspects of their lives (e.g., physical health, spirituality), self-forgiveness has been relatively neglected in scientific research on forgiveness. In fact, Hall and Fincham (2005) once called self-forgiveness the “stepchild of the forgiveness literature” (p. 621). Although self-forgiveness research has since made substantial advances both empirically and theoretically (for an overview, see Woodyatt et al., 2017; Woodyatt and Wenzel, 2020), there are still various topics for which much less is known about self-forgiveness than about interpersonal forgiveness.

In this article, we addressed one such topic for which there still exists a substantial knowledge gap between the two types of forgiveness: the personality traits of forgiving individuals. Specifically, we report an individual differences study in which we used the same set of transgression scenarios of the kind described above for both types of forgiveness and examined whether (and how) the personality correlates of self-forgiveness differed from those of interpersonal forgiveness. As a secondary, more exploratory aim, we also examined whether our scenario-based assessment of forgiveness differed from a questionnaire-based assessment of trait forgiveness, using a widely used measure, the Self and Other subscales of the Heartland Forgiveness Scale (HFS; Thompson and Snyder, 2003; Thompson et al., 2005). To the best of our knowledge, this is the only study that examined the personality correlates of dispositional interpersonal and self-forgiveness by directly comparing them within a single study, using multiple assessment methods.

### 1.1. Motivational-change framework for interpersonal and self-forgiveness

Although different definitions and conceptualizations of forgiveness exist (for reviews, see Tucker et al., 2015; Worthington, 2020), the current research adopted, as the theoretical framework, a *motivational-change* view of forgiveness proposed by McCullough et al. (1998): “a complex of prosocial changes in one’s interpersonal motivations following a transgression” (McCullough and Hoyt, 2002, p. 1556). According to this view, forgiveness can be construed as changes in three transgression-related motivations toward the transgressors: (a) motivations to avoid them (*avoidanc*e), (b) motivations to seek revenge (*revenge*), and (c) motivations to show goodwill toward them (*benevolence*).

Although this multicomponent view was developed primarily for characterizing interpersonal forgiveness, it also provides a useful basis for conceptualizing self-forgiveness. Based on McCullough et al. (1998) framework, Hall and Fincham (2005) defined self-forgiveness as “a set of motivational changes whereby one becomes decreasingly motivated to avoid stimuli associated with the offense, decreasingly motivated to retaliate against the self [.], and increasingly motivated to act benevolently toward the self” (p. 622), thus incorporating the same three transgression-related motivations of avoidance, revenge, and benevolence, respectively, into their definition.

Although there are newer and broader-scope conceptions of self-forgiveness that include additional components such as accountability, human connectedness, and commitment to change (see Webb et al., 2017, for a review), we adopted this simpler conception of self-forgiveness by Hall and Fincham (2005) for two reasons. First, it still fits with the general growing agreement in the field that self-forgiveness includes two key components: (a) accepting responsibility for wrongdoing and (b) restoring one’s sense of self (e.g., Woodyatt and Wenzel, 2013, 2020; Webb et al., 2017; Cornish et al., 2018). Specifically, the former can be construed as having lower levels of avoidance motivations and higher levels of benevolence motivations, and the latter as having lower levels of revenge (or self-punishing) motivations. Second and more directly relevant to the current study, by adopting these parallel definitions of interpersonal and self-forgiveness and assessing the same three transgression-related motivations, we wanted to make the comparison between the two types of forgiveness as direct as possible within a single study.

Important to note, however, we made one modification to Hall and Fincham’s (2005) definition of self-forgiveness quoted above: In their definition, the target of benevolent actions is the transgressor (the self), but we operationalized benevolence for self-forgiveness as motivations to show goodwill toward the victim of the transgression, not the self. We adopted a different conceptualization here because existing conceptions of self-forgiveness also postulate that an act of seeking reconciliation and restoring the relationship with the victim is an important process for achieving high levels of self-forgiveness (e.g., Rangganadhan and Todorov, 2010; McConnell et al., 2012; Webb et al., 2017; Cornish et al., 2018). Thus, by designating the victim, not the transgressor (the self), as the target of benevolent actions, our conceptualization better represents this conciliatory effort associated with benevolence motivations for self-forgiveness.

### 1.2. Personality correlates of forgiveness

The main goal of the current study was to examine whether the personality correlates of self-forgiveness differ systematically from those of interpersonal forgiveness at the dispositional level. Focusing on the Big Five model of personality (McCrae and John, 1992), previous research has yielded consistent patterns for interpersonal forgiveness. Specifically, *agreeableness* and *neuroticism* have been the two Big Five factors most consistently associated with measures of dispositional interpersonal forgiveness, as revealed by recent systematic (Fernández-Capo et al., 2017) and meta-analytic (Mullet et al., 2005; Balliet, 2010; Hodge et al., 2020) reviews. For example, the most recent meta-analysis by Hodge et al.’s (2020) reported that, for dispositional interpersonal forgiveness, the aggregate effect sizes were substantial for agreeableness, *r* = 0.44 (*k* = 52), and neuroticism, *r* = –0.28 (*k* = 52), but were considerably smaller for conscientiousness, *r* = 0.12 (*k* = 42), extraversion, *r* = 0.15 (*k* = 43), and openness to experience, *r* = 0.11 (*k* = 42).

Although many of the studies included in the meta-analysis were based on questionnaire data, a smaller number of interpersonal forgiveness studies using different assessment methods have yielded similar results. For example, using a scenario-based method, McCullough and Hoyt (2002) assessed, in two studies, three transgression-related motivations (avoidance, revenge, and benevolence) for interpersonal forgiveness. In both studies, agreeableness and neuroticism were the two Big Five factors that consistently predicted the three transgression-related motivations. More specifically, agreeableness was associated with revenge, suggesting that more agreeable individuals had weaker revenge motivations toward transgressors. In contrast, neuroticism was associated positively with avoidance and negatively with benevolence, suggesting that more neurotic individuals had stronger avoidance motivations but weaker benevolence motivations. Similarly, using a scenario-based assessment tool called the Transgression Narrative Test of Forgiveness (TNTF), Berry et al. (2001) also found that agreeableness and neuroticism predicted interpersonal forgiveness across multiple studies.

In contrast to such consistent patterns of results for interpersonal forgiveness, our understanding of the personality correlates of self-forgiveness is more limited because the number of existing studies is considerably smaller. Moreover, most prior studies that examined the personality correlates of self-forgiveness adopted a questionnaire-based approach (e.g., using the HFS’s Self subscale). Other assessment approaches, especially the scenario-based approach used in interpersonal forgiveness research (e.g., Berry et al., 2001; McCullough and Hoyt, 2002), are rare in the investigation of the personality correlates of self-forgiveness.

Even among the questionnaire-based studies, the patterns of correlations are not necessarily consistent across studies. For example, two recent studies (Oral and Arslan, 2017; Walker, 2017) used the Self subscale of the HFS as the measure of dispositional self-forgiveness and assessed personality factors. In addition to the Big Five factors, Walker (2017) included a measure of grit (Duckworth et al., 2007), whereas Oral and Arslan (2017) included measures of self-compassion (Neff, 2003) and ruminative tendencies for interpersonal offenses (Wade et al., 2008). Regression results indicated that the Self subscale of the HFS was uniquely predicted by only grit and neuroticism in the Walker (2017) study and by only self-compassion and extraversion in the Oral and Arslan (2017) study. Such differences likely reflect different additional non-Big-Five variables included in the respective studies^1^ as well as potential cultural (religious) differences between the two samples (American vs. Turkish). Nevertheless, it is reasonable to suggest that these similar studies based on the same Self subscale of the HFS did not yield fully convergent results.

Hodge et al.’s (2020) aforementioned meta-analytic review also examined the personality correlates of dispositional self-forgiveness and reported the following effect size estimates based on a substantially fewer number of studies (*k*’s = 13–15 vs. *k*’s = 42–52 for interpersonal forgiveness): *r* = 0.28 (*k* = 15) for agreeableness, *r* = –0.40 (*k* = 18) for neuroticism, *r* = 0.19 (*k* = 13) for conscientiousness, *r* = 0.25 (*k* = 15) for extraversion, and *r* = 0.13 (*k* = 13) for openness to experience. These effect-size estimates are consistent with the findings of Oral and Arslan’s (2017) and Walker’s (2017) studies in some respects (i.e., substantial correlations with neuroticism and extraversion, respectively), but, at the same time, they are also different from those for dispositional interpersonal forgiveness (e.g., correlations with agreeableness were substantially weaker for self-forgiveness than for interpersonal forgiveness).

Clearly, more work is needed to better establish the personality correlates of self-forgiveness. In fact, in the discussion of their meta-analytic results, Hodge et al. (2020) explicitly pointed out “the relative dearth of empirical research on self-forgiveness compared to other-forgiveness” and strongly “encourage[d] researchers to continue the study of self-forgiveness to better understand its effects on well-being and mental health outcomes” (p. 103). In our view, a need for further studying the personality correlates of self-forgiveness is strong, given that most (if not all) of the existing studies on this topic used questionnaires based on self-ratings on general decontextualized statements (e.g., Walker and Gorsuch, 2002; Leach and Lark, 2004; Ross et al., 2004; Oral and Arslan, 2017; Walker, 2017). We therefore reasoned that a study using a different way of assessing self-forgiveness, such as a scenario-based method, would make a unique contribution to the literature.

### 1.3. The current study

#### 1.3.1. Our approach

The primary goal of the current study was to examine the personality correlates of dispositional self-forgiveness and directly compare them against those of interpersonal forgiveness within a single study. Toward this goal, we used a scenario-based approach used effectively before to examine the personality correlates of interpersonal forgiveness (e.g., Berry et al., 2001; McCullough and Hoyt, 2002).

An example scenario is provided in Table 1. Except for whether the reader is the transgressor or the victim, the scenarios were essentially the same for the two versions (the differences are indicated in italics in Table 1). In line with the motivational-change view of forgiveness (McCullough and Hoyt, 2002; Hall and Fincham, 2005), we assessed three transgression-related motivations (avoidance, revenge, and benevolence). In addition, as was done in the TNTF (Berry et al., 2001), we also assessed global levels of forgiveness.

**TABLE 1:** Sample scenario and forgiveness-assessment items.

Interpersonal forgiveness scenario	Self-forgiveness scenario
*You have* been saving up *your* vacation days for the entire year, and *you* finally decide to take 2 weeks and go on *your* dream vacation. The only problem is that *you* have a dog and cannot leave it alone for so long. Putting *your* dog in a kennel for 2 weeks is not only too expensive, but *you* care about your dog too much to leave it in such a place. *One of your friends offers* to watch your dog. *She* works from home so the dog will get plenty of attention. *You* are very relieved that this problem is resolved, and *you* proceed to take *your* dream vacation. Before *you go*, *you remind your friend* to make sure not to leave any doors open, for *your* dog likes to get outside whenever it can. *Your* vacation goes very well. The day *you* come to pick up *your* dog, *your friend goes* to the grocery store and accidently leaves the front door ajar. *You come* to pick up the dog before *your friend gets* back, and *you* find it on the road; it was hit by a car.	*Your friend has* been saving up *her* vacation days for the entire year, and *she* finally decides to take 2 weeks and go on *her* dream vacation. The only problem is that *she* has a dog and cannot leave it alone for so long. Putting *her* dog in a kennel for 2 weeks is not only too expensive, but *she* cares about the dog too much to leave it in such a place. *You offer* to watch the dog. *You* work from home, so the dog will get plenty of attention. *Your friend* is very relieved that this problem is resolved, and *she* proceeds to take *her* dream vacation. Before *she goes*, *she reminds you* to make sure not to leave any doors open, for *her* dog likes to get outside whenever it can. *Your friend’s* vacation goes very well. The day *she* comes to pick up *her* dog, *you go* to the grocery store and leave the front door ajar. *Your friend comes* to pick up the dog before *you get* back, and *she* finds it on the road; it was hit by a car.
1. How upset would you be if this happened to you? (Offense Severity)	1. How upset would you be if you did this? (Offense Severity)
2. I recently experienced a similar situation. (Recent Experience)	2. I recently experienced a similar situation. (Recent Experience)
3. I would be trying to keep as much distance between us as possible. (Avoidance)	3. I would be trying to keep as much distance between us as possible. (Avoidance)
4. I would want to make her pay. (Revenge)	4. I should have to pay for what I did. (Revenge)
5. I would be striving to achieve reconciliation. (Benevolence)	5. I would be striving to achieve reconciliation. (Benevolence)
6. I would never be able to forgive her. (Eventual: R)	6. I would never be able to forgive myself. (Eventual: R)
7. How long would it take you to forgive her? (Time: R)	7. How long would it take you to forgive yourself? (Time: R)

Differences between conditions are italicized. Not included here are three additional items measuring avoidance, revenge, and benevolence. The full set of scenarios and forgiveness assessment items are available in the Supplementary Material A. R, reverse-coded.

Although there are various ways to assess interpersonal forgiveness and self-forgiveness (for reviews, see Fernández-Capo et al., 2017; Strelan, 2017; McElroy-Heltzel et al., 2020), we adopted this scenario-based approach because, unlike other approaches (e.g., self-ratings on existing questionnaires, recalling and rating recent personally experienced episodes requiring forgiveness), it allowed us to make the transgression-related scenarios equivalent for both interpersonal and self-forgiveness. In other words, we were able to “manipulate” the two types of forgiveness within the same study, which has rarely been done in the literature (for a notable exception, see Leunissen et al., 2013, although their focus was not on the personality correlates of forgiveness). Moreover, our focus on the same transgression-related motivations (avoidance, revenge, and benevolence) also allowed us to assess the two types of forgiveness in a more directly comparable manner than might otherwise be possible with other methods.

The scenarios we created, however, differed from those used in previous studies of interpersonal forgiveness by McCullough and Hoyt (2002) in one important way: Instead of intentionally varying some key features of the target transgressions across scenarios (e.g., the nature of the relationship between the transgressor and the victim), we tried to minimize such situational variability across scenarios to reduce sources of across-scenario variability. For example, in each scenario, the victim or the transgressor of the described offense was somebody close to the reader. Each scenario emphasized that the future victim gave the future transgressor an advance warning about potential problems that might arise, but that the transgressor nonetheless failed to heed that warning, thus making the target of the blame (“who dropped the ball”) clear. Moreover, the transgressions described in the scenarios are severe and highly upsetting to most readers.

Such relative homogeneity of the scenarios would not be ideal if the goal of the study were to investigate how these different situational factors differentially affect the levels of interpersonal forgiveness versus self-forgiveness. Given that the goal of our study was to examine the personality correlates of forgiving individuals, rather than situational factors affecting forgiveness, our priority was instead to minimize across-scenario variability and obtain stable estimates of individual differences in forgiveness that are consistently observed across scenarios. Although doing so would limit the generalizability of the results, it would require fewer scenarios to obtain reliable estimates of individual differences in interpersonal and self-forgiveness. As a first scenario-based study seeking to directly compare the personality correlates of two types of forgiveness, we decided that using relatively homogeneously structured scenarios would be justifiable.

In addition to such scenario-based assessment of forgiveness, we also assessed the Big Five factors and explanatory style. Explanatory style concerns the ways in which people tend to interpret the causes of personally relevant events, especially those involving failure (Abramson et al., 1978; Seligman, 1990). Individuals with pessimistic explanatory styles tend to internalize the negative events and blame themselves, whereas those with optimistic styles tend to attribute failures to external sources. Although explanatory style has not been used in prior forgiveness research, we included it here because one component of this construct, the personal component (the tendency to internalize the failure), seemed highly relevant to one’s willingness to forgive oneself.

#### 1.3.2. Main aims of the study and predictions

The primary aim of this study was to examine whether the personality correlates of forgiveness differed for interpersonal and self-forgiveness. As reviewed earlier, prior research has shown that agreeableness and neuroticism are the two Big Five factors consistently associated with interpersonal forgiveness. Moreover, McCullough and Hoyt (2002) observed unique associations of agreeableness to revenge motivations and of neuroticism to avoidance and benevolence motivations. We thus expected similar patterns of results for our interpersonal forgiveness scenarios.

The predictions for the self-forgiveness scenarios were less clear, given the relative paucity and inconsistency of prior evidence, as reviewed earlier. Because the self-forgiveness and interpersonal forgiveness are unlikely to be totally independent (in fact, the HFS Self and Other subscales typically correlate at 0.30–0.35; Thompson et al., 2005), we expected agreeableness and neuroticism to be likely correlates for self-forgiveness as well. However, we also hypothesized two additional correlates for our scenario-based measures of self-forgiveness: conscientiousness and explanatory style. Specifically, we hypothesized that people high in conscientiousness—a trait associated with attributes like “reliable,” “dutiful,” and “responsible”—would likely have a particularly hard time forgiving themselves for their own negligence. We also hypothesized that individuals who tend to attribute the failure to internal sources and blame themselves (the personal component of explanatory style) would have greater difficulty forgiving themselves.

A secondary, more exploratory aim of the study was to compare a scenario-based assessment of dispositional forgiveness to a more commonly used questionnaire-based assessment. Following some previous studies reviewed above (Oral and Arslan, 2017; Walker, 2017), we used the Self and Other subscales of the HFS as the target forgiveness questionnaire. Given that the HFS assesses dispositional forgiveness based on general decontextualized statements, we expected some differences in the personality correlates of forgiveness between these two assessment methods, but we did not have specific *a priori* hypotheses about the nature of differences between them. However, on the plausible assumption that the scenario-based and questionnaire-based assessments of forgiveness seek to capture the same underlying constructs (i.e., interpersonal and self-forgiveness), we expected that the Other subscale of the HFS would be more strongly associated with interpersonal forgiveness than with self-forgiveness as assessed with our transgression scenarios. Similarly, we also expected that the Self subscale of the HFS would be more strongly associated with self-forgiveness than with interpersonal forgiveness as assessed with our transgression scenarios.

## 2. Materials and methods

The current study was not preregistered. The stimulus materials used in the study are available as an online Supplementary Material A. The data and data-analytic codes (in R) are available at the project’s Open Science Framework (OSF) site.^2^

### 2.1. Participants

One hundred seventy students from an introductory psychology course, all native speakers of English or highly fluent in English, participated for partial course credit. They were randomly assigned to either the interpersonal forgiveness condition or the self-forgiveness condition (*n*’s = 85 and 85, respectively). We used a between-subjects design to avoid presenting both versions of the same scenario to participants and thereby eliminate possible carry-over effects across two different types of scenarios (e.g., responding to an interpersonal forgiveness scenario may influence the same participant’s responding to a self-forgiveness scenario).

Of the 170 participants, we excluded 10 participants from analysis for the following predetermined reasons: (a) incorrectly answering both of the two “catch” questions included in the questionnaires to assess random or inattentive responding (*n*s = 1 and 3, respectively)^3^ or (b) due to experimental error, such as incorrect condition assignment (*n*s = 2 and 4). Thus, the final sample size was 160 (*n* = 82 in the interpersonal forgiveness condition and *n* = 78 in the self-forgiveness condition). The demographic characteristics of the participants in the two conditions were generally comparable in terms of gender, age, and race/ethnicity.^4^

### 2.2. Sample size and power considerations

The current study was conducted as part of the first author’s undergraduate honors thesis. Due to the limited time available for designing, implementing, and pilot-testing the study and collecting and analyzing the data for this thesis project, the achievable sample size was limited, especially because we tested participants individually in person (see section “2.3. Overall procedure” below for further detail). Moreover, because our scenarios were newly developed for this study, we did not know how they would correlate with personality measures, thus making it difficult for us to conduct a well-informed *a priori* power analysis to determine our sample size.

For these reasons, although our intention was to collect data from as many participants possible within the limited timeframe, we aimed for collecting data from 100 participants in each of the two forgiveness conditions (for a total of 200 participants). According to a *post hoc* power analysis we conducted using G*Power, with a sample size of 100, a correlation of *r* = 0.276 would have been needed to achieve a statistical power of 0.80, although the critical value for a correlation to be significant for that sample size was *r* = 0.196.

Because we failed to reach that target sample size, we conducted an additional *post hoc* power analysis. For the sample sizes of 82 (interpersonal) and 78 (self), the correlations as large as *r* = 0.303 and *r* = 0.311, respectively, are needed to achieve a statistical power of 0.80, but the critical values were 0.217 and 0.223. In light of the effect size estimates reported in Hodge et al.’s (2020) meta-analysis described earlier (see section “1.2. Personality correlates of forgiveness”), these sample sizes were likely sufficient for at least detecting the bivariate correlations that we predicted to be present (i.e., agreeableness and neuroticism for both types of forgiveness and conscientiousness for self-forgiveness, with their effect size estimates of *r* = 0.25 or greater), albeit not necessarily with a 0.80 power.

### 2.3. Overall procedure

Participants were tested individually in person in a quiet room. After providing consent and completing a brief demographic questionnaire, all participants completed three questionnaires, all administered using Qualtrics, in the following order: explanatory style, general dispositional forgiveness (HFS), and personality (Big Five).

All participants then performed two computerized measures of working memory capacity, called reading span and spatial span, to assess participants’ executive-control ability (for further details, see Shah and Miyake, 1996; Miyake et al., 2001). These tasks required participants to maintain target information (words or letter orientations) while processing information (verifying sentences or performing mental rotations). We included these measures in this study because some prior reports have suggested that executive-control ability may be associated with forgiveness (Burnette et al., 2014) or serve as a moderator (Pronk et al., 2010). We will not discuss these measures in this article any further, however, because they did not significantly correlate with any of the other measures included in the study.^5^

Finally, participants read six forgiveness scenarios in the same order (see Appendix A) and, for each scenario, responded to a set of forgiveness assessment questions. We administered our scenario-based assessment of forgiveness last to minimize the likelihood that responding to forgiveness scenarios would affect participants’ responses to the other individual differences measures (especially, the HFS). The entire testing session lasted about 50 min.

### 2.4. Individual differences measures

#### 2.4.1. Personality

We used the Big Five Inventory (BFI; John et al., 1991) to assess the five dimensions of personality: agreeableness, conscientiousness, neuroticism, extraversion, and openness to experience. This 44-item questionnaire asks participants to rate, on a 5-point Likert scale, the extent to which each short phrase (e.g., *is a reliable worker* for conscientiousness) accurately describes them.

#### 2.4.2. Explanatory style

Explanatory style was assessed with the Expanded Attributional Style Questionnaire–Short (EASQ–S; Whitley, 1991). This questionnaire asks the participant to generate a cause to each of the hypothetical negative situations (e.g., one might generate a possible cause, “I’m careless and make a lot of mistakes,” to the prompt, *You are fired from your job*). The participant is then asked to respond, using a 7-point Likert scale, to three questions about the cause they assigned, each targeting the locus, stability, or globality of the cause (corresponding to the personal, permanent, and pervasive components of explanatory style, respectively). Although the EASQ–S includes 12 negative-event prompts, we used 9 of them due to time constraints.

We focused our analysis on the personal component (the tendency to attribute the cause of the negative event to oneself), which we hypothesized should be particularly relevant to self-forgiveness. Higher scores of the personal component of explanatory style indicated a higher tendency to attribute the failure to external, rather than internal, sources.

#### 2.4.3. General dispositional forgiveness

The HFS (Thompson and Snyder, 2003) is an 18-item questionnaire designed to assess self, other, and situational forgiveness by asking participants to “think about how [they] would typically respond to negative events.” Each subscale consists of 6 questions on a 7-point Likert scale, with the average taken as the final score. We administered only the Self and Other subscales (12 items in total) in this study, not the Situations subscale.

### 2.5. Narrative-based assessment of dispositional interpersonal and self-forgiveness

We created six transgression scenarios, including the “pet death” scenario shown in Table 1, to assess dispositional interpersonal and self-forgiveness (see Supplementary Material A for all the scenarios used in this study). We modeled these scenarios primarily after those created by Berry et al. (2001) for their Transgression Narrative Test of Forgiveness (TNTF) scale.

Each scenario involved two protagonists, “you” (the reader) and the “other” person with whom the reader has a close relationship of some sort (e.g., a good friend), and always ended with severe negative consequences (e.g., a pet death, car theft, property loss). The two versions of each scenario differed only in terms of who was the transgressor versus the victim. All six narratives clearly indicated that the future victim had some concerns (e.g., the dog likes to sneak out) and warned the future transgressor about it, but that the transgressor failed to heed that warning, thus directly contributing to the tragedy.

Following each scenario was a set of 10 items eliciting background ratings about the scenario (2 items) and assessing the levels of forgiveness (8 items). The first background item (Offense Severity) concerned the perceived severity of the transgression, assessed on a 5-point Likert scale (1 = not upset at all; 5 = extremely upset), and the second background item (Recent Experience) concerned the participant’s recent experience of similar situations, rated on a 6-point Likert scale (1 = strongly disagree; 6 = strongly agree). These two background items were included to check our basic assumptions about our transgression scenarios, namely that the events described in our scenarios are severe and evoke strong emotional reactions (Offense Severity) but are rare enough that most participants had not experienced something similar recently (Recent Experience).

The next 6 items, which assessed the levels of three transgression-related motivations, were adopted from the Transgression-Related Interpersonal Motivations Scale (McCullough et al., 1998). The items selected for interpersonal forgiveness were essentially the same as those used in the original scale, modified only to match the gender of the transgressor in each scenario. For the self-forgiveness items, however, additional changes were made so that the target of avoidance and benevolence motivations would always be the other party (the victim), and that the target of revenge motivations would always be “you” (the transgressor). Each transgression-related motivation was assessed with two items, all on a 6-point Likert scale (1 = strongly disagree; 6 = strongly agree). For brevity, Table 1 lists only a subset of these items, but the online Supplementary Material A provides a complete list of all items for each scenario.

The last two items assessed the global level of forgiveness. The first item—the eventual likelihood of forgiveness (Eventual)—assessed, on a 6-point Likert scale (1 = strongly disagree; 6 = strongly agree), how likely the participant would ultimately forgive the transgressor or oneself. The second item (Time), on an 8-point nominal scale, assessed how long it would take the participant to forgive the transgressor.^6^ The original scales for these global forgiveness items were such that higher ratings meant lower levels of forgiveness, but we reverse-coded both items for ease of interpretation so that higher numbers meant higher levels of forgiveness.

To derive final scores, we first averaged each participant’s responses to two items for each forgiveness measure (avoidance, revenge, benevolence, and global) within each scenario. We then aggregated those mean ratings by averaging them across six scenarios.

## 3. Results

### 3.1. Preliminary analyses

All of the statistical analyses were conducted in R. For transparency and reproducibility, the data-analytic scripts for all of the analyses reported in this article (as well as the resulting statistical outputs) are available at the project’s OSF site (see text footnote 2).

#### 3.1.1. Forgiveness measures: descriptive statistics

The descriptive statistics for the scenario-based measures of interpersonal and self-forgiveness (means [*SD*s], range, and reliability estimates) are summarized in Table 2. All the measures had satisfactory reliabilities across the scenarios in both conditions (Cronbach’s α ranging from 0.70 to 0.91 in the interpersonal forgiveness condition and from 0.75 to 0.92 in the self-forgiveness condition).

**TABLE 2:** Descriptive statistics for the forgiveness measures in the interpersonal and self-forgiveness conditions (*N* = 160).

	Interpersonal forgiveness (*n* = 82)			Self-forgiveness (*n* = 78)			Differences
**Measures**	***M (SD)***	**Range**	**α**	***M (SD)***	**Range**	**α**	***t* (*p*)**
**Background ratings**							
Offense severity	4.38 (0.42)	3.00–5.00	0.75	4.40 (0.47)	2.83–5.00	0.75	−0.36 (0.716)
Recent experience	1.70 (0.71)	1.00–4.00	0.74	1.51 (0.72)	1.00–3.50	0.84	***1.71* (0.089)**
**Transgression-related motivations**							
Avoidance	3.68 (0.77)	1.92–5.58	0.86	2.83 (0.93)	1.00–4.92	0.92	**6.24 (<0.001)**
Revenge	3.24 (1.01)	1.00–5.58	0.91	4.71 (0.82)	1.92–6.00	0.89	−**10.14 (<0.001)**
Benevolence	3.37 (0.62)	1.83–5.17	0.78	4.85 (0.61)	3.58–6.00	0.83	−**15.25 (<0.001)**
Global forgiveness	0.30 (0.77)	−1.57–1.92	—	−0.32 (0.94)	−2.68–1.22	—	—
Eventual	4.20 (0.82)	2.50–5.67	0.70	3.37 (1.00)	1.17–5.50	0.83	**5.71 (<0.001)**
Time	4.83 (0.99)	2.33–6.83	0.74	4.37 (1.23)	1.33–6.67	0.85	**2.61 (0.010)**

Bolded values indicate *p* < 0.05, whereas italics indicate 0.10 > *p* > 0.05. Eventual forgiveness is reverse coded such that higher values indicate greater forgiveness. Global forgiveness is a z-score aggregate of Eventual and Time components (both reverse-coded). α = Cronbach’s alpha.

##### 3.1.1.1. Background measures

As expected, the Offense Severity ratings were high (*M*’s = 4.38 and 4.40 out of 5, respectively), suggesting that they would be seriously upset if they were implicated in those situations, whether as the victim or as the transgressor. Also, the Recent Experience ratings were low (*M*’s = 1.70 and 1.51 out of 6 for interpersonal and self-forgiveness, respectively), suggesting that they had not recently experienced tragic events like the ones described in the scenarios. These results were consistent with our basic assumption about the events described in the scenarios (i.e., the scenarios describe severe yet rare transgressions).

##### 3.1.1.2. Three transgression-related motivations

More important are the forgiveness ratings for the three transgression-related motivations, for which higher numbers meant having stronger avoidance, revenge, and benevolence motivations, respectively (all assessed on a 6-point Likert scale). Interesting to note, the mean ratings for each motivation differed significantly between the interpersonal and self-forgiveness conditions, as revealed by the independent-group *t*-tests reported in Table 2.

Specifically, participants were more likely to avoid the other party if they were the victims (interpersonal) than if they were the transgressors (self). In contrast, their revenge motivations were stronger for self-forgiveness situations than for interpersonal forgiveness situations, suggesting that, even for the transgression of equivalent severity, the tendency to blame themselves and feel that “I should pay” is stronger when they were the transgressors than the tendency to blame the other party and feel that “he/she should pay” when they were the victims. Finally, the motivation to act benevolently toward the victim was stronger if the participants were the transgressors (self) than if they were victims (interpersonal).

##### 3.1.1.3. Global forgiveness

We assessed global levels of forgiveness by asking about the ultimate likelihood of forgiving (Eventual) and the time it would take to forgive the transgressor (Time), with higher scores indicating greater levels of forgiveness. As shown in Table 2, the likelihood of eventual forgiveness was higher if the transgressor was the other party (interpersonal) than themselves (self). Similarly, participants in the interpersonal forgiveness condition also thought that forgiveness would likely take less time than those in the self-forgiveness condition.

#### 3.1.2. Forgiveness measures: bivariate correlations

The intercorrelations among the measures discussed above (except for Recent Experience) are summarized in Table 3, separately for the two forgiveness conditions. Consistent with some earlier reports (e.g., Barcaccia et al., 2020), the three transgression-related motivations were substantially correlated with one another in the interpersonal forgiveness condition, as shown in the left panel (*r*’s = 0.42 between avoidance and revenge, –0.39 between avoidance and benevolence, and –0.26 between avoidance and benevolence).

**TABLE 3:** Correlations among the forgiveness measures in the interpersonal and self-forgiveness conditions (*N* = 160).

	Interpersonal forgiveness (*n* = 82)					Self-forgiveness (*n* = 78)				
**Measure**	**1.**	**2.**	**3.**	**4.**	**5.**	**1.**	**2.**	**3.**	**4.**	**5.**
1. Offense severity	–					–				
2. Avoidance	**0.33**	–				−**0.24**	–			
3. Revenge	0.12	**0.42**	–			**0.53**	−0.02	–		
4. Benevolence	−**0.26**	−**0.39**	−**0.26**	–		**0.37**	−**0.45**	**0.26**	–	
5. Global	−***0.20***	−**0.39**	−**0.62**	**0.54**	–	−**0.41**	−0.02	−**0.47**	−***0.19***	–

Bolded values indicate *p* < 0.05, whereas bold italics indicate 0.10 > *p* > 0.05.

Interestingly, the results were somewhat different in the self-forgiveness condition, as shown in the right panel. Although benevolence motivations correlated with the other two transgression-related motivations (*r*’s = –0.45 with avoidance and 0.26 with revenge), there was no correlation between revenge and benevolence (*r* = 0.02). A follow-up Fisher’s *r*-to-*z* transformation test, which we used to compare the magnitudes of the correlations observed between avoidance and revenge, demonstrated that it was significantly higher in the interpersonal forgiveness condition (*r* = 0.42) than in the self-forgiveness condition (*r* = –0.02), *z* = 2.89, *p* = 0.004. This finding will be considered later in the Discussion section (see section “4.1.2. Different patterns of correlations among three transgression-related motivations”).

#### 3.1.3. Individual differences measures

The descriptive statistics for the personality and explanatory style measures as well as the HFS Other and Self subscales (means [*SD*s], ranges, and reliability estimates) are provided in Table 4. All the measures summarized in this table had satisfactory Cronbach’s alphas (in the 0.70 to 0.87 range), except for the personality component of explanatory style (α = 0.27).^7^

**TABLE 4:** Descriptive statistics and correlations for the individual differences measures for the full sample (*N* = 160).

	Descriptive statistics			Correlations							
**Measure**	***M (SD)***	**Range**	**α**	**1**	**2**	**3**	**4**	**5**	**6**	**7**	**8**
1. Agreeableness	3.73 (0.49)	2.22–4.89	0.70	–							
2. Neuroticism	3.02 (0.67)	1.38–4.62	0.82	−**0.22**	–						
3. Conscientiousness	3.48 (0.58)	1.56–4.89	0.79	**0.40**	−0.08	–					
4. Openness	3.66 (0.47)	2.20–4.80	0.72	**0.20**	−***0.14***	−0.05	–				
5. Extraversion	3.23 (0.76)	1.12–5.00	0.87	**0.18**	−**0.16**	***0.14***	**0.25**	–			
6. EASQ-S (personal)	4.86 (0.66)	3.00–6.56	0.27	−0.02	−0.10	0.00	0.04	−0.08	–		
7. HFS (other)	4.78 (0.91)	2.67–7.00	0.77	**0.50**	−***0.14***	***0.13***	**0.18**	0.11	0.06	–	
8. HFS (Self)	4.73 (1.02)	1.50–7.00	0.79	**0.31**	−**0.54**	0.09	**0.30**	**0.34**	0.09	**0.21**	–

Bolded values indicate *p* < 0.05, whereas bold italics indicate 0.10 > *p* > 0.05. EASQ-S (Personal) = the personal component of the Expanded Attributional Style Questionnaire–Short. HFS, Heartland Forgiveness Scale. α = Cronbach’s alpha.

Table 4 also reports the intercorrelations among them. Because participants completed these measures before they were randomly assigned to one of the two forgiveness conditions, the results shown here are for the entire sample (*N* = 160).^8^ Most of the correlational results were expected, but one finding worth noting here is that the personal component of explanatory style was not correlated with any other measures listed in this table. This lack of correlations likely reflects in part the low internal reliability of this measure for this sample, as noted above.

### 3.2. Primary analyses: personality correlates of forgiveness

#### 3.2.1. Bivariate correlations

The primary aim of this study was to directly compare the personality correlates of interpersonal and self-forgiveness within a single study using a scenario-based assessment method. The top portion of Table 5 (rows 1–6) reports the correlations between the individual-differences measures (the Big Five factors and the personal component of explanatory style) administered in this study and our four measures of forgiveness (avoidance, revenge, benevolence, and global).

**TABLE 5:** Correlations between personality variables and the scenario-based forgiveness measures (*N* = 160).

	Interpersonal forgiveness (*n* = 82)				Self-forgiveness (*n* = 78)			
**Measure**	**Avoidance**	**Revenge**	**Benevolence**	**Global**	**Avoidance**	**Revenge**	**Benevolence**	**Global**
1. Agreeableness	−0.04	−**0.48**	0.16	**0.31**	−**0.28**	*0.22*	*0.20*	−*0.20*
2. Neuroticism	**0.23**	***0.19***	−***0.19***	−**0.26**	**0.28**	0.13	−0.06	−**0.27**
3. Conscientiousness	0.06	−***0.21***	0.04	0.06	−**0.34**	0.05	0.18	−**0.35**
4. Openness	−0.15	−0.18	−0.02	0.18	−0.10	0.05	−0.07	0.06
5. Extraversion	−0.12	−0.18	0.17	0.12	−0.13	0.13	0.11	−0.15
6. EASQ–S (personal)	0.11	0.07	0.06	−0.06	−**0.26**	0.03	−0.01	0.08
7. HFS (other)	−**0.32**	−**0.48**	**0.27**	**0.45**	−**0.28**	−0.01	0.02	0.03
8. HFS (self)	−**0.25**	−**0.29**	0.17	**0.34**	−**0.30**	−0.17	0.08	0.15

Bolded values indicate *p* < 0.05, whereas bold italics indicate marginal significance 0.10 > *p* > 0.05. EASQ–S (Personal) = the personal component of the Expanded Attributional Style Questionnaire–Short. HFS, Heartland Forgiveness Scale.

As hypothesized, in the interpersonal forgiveness condition (the left panel), agreeableness and neuroticism were the two Big Five dimensions that consistently correlated with the forgiveness measures, including the global measure (*r* = 0.31 and –0.26, respectively). Consistent with McCullough and Hoyt (2002), neuroticism was associated positively with avoidance motivations (*r* = 0.23) and negatively (albeit marginally) with benevolence motivations (*r* = –0.19), whereas agreeableness was associated negatively with revenge motivations (*r* = –0.48). The correlation patterns for self-forgiveness (the right panel of Table 5) were similar in that agreeableness and neuroticism were also associated with forgiveness, especially avoidance motivations (*r* = –0.28 and 0.28, respectively) and global forgiveness (*r* = –0.20 and –0.27).

Important to note, however, there were two differences consistent with our predictions for the self-forgiveness condition. First, conscientiousness was a reliable correlate of global forgiveness (*r* = –0.35) as well as avoidance motivations (*r* = –0.34), suggesting that more conscientious individuals have greater difficulty forgiving themselves when they are aware that their negligence led to the tragic outcome that they could have avoided. Second, although the association was restricted only to avoidance motivations, the personal component of explanatory style was negatively correlated with self-forgiveness (*r* = –0.26), indicating that individuals who internalize their failures tend to have stronger motivations to avoid the victim.

These unique associations of conscientiousness and explanatory style with self-forgiveness, but not with interpersonal forgiveness, were substantiated by follow-up Fisher’s *r*-to-*z* transformation tests. As hypothesized, conscientiousness was more strongly correlated with global forgiveness in the self-forgiveness condition (*r* = –0.35) than in the interpersonal forgiveness condition (*r* = 0.06), *z* = 2.63, *p* = 0.009. Likewise, its correlation with avoidance motivations was stronger in the self-forgiveness condition (*r* = –0.34) than in the interpersonal forgiveness condition (*r* = 0.06), *z* = 2.56, *p* = 0.011. Finally, explanatory style was also a stronger correlate of avoidance motivations in the self-forgiveness condition (*r* = –0.26) than in the interpersonal forgiveness condition (*r* = 0.11), *z* = 2.31, *p* = 0.021. These results suggest that, at least for the types of situations depicted in the scenarios used in the current study, conscientiousness and explanatory style (the personal component) matter more for self-forgiveness than for interpersonal forgiveness.

#### 3.2.2. Multiple regressions

We next conducted multiple regression analyses to examine which of these personality correlates were unique predictors of each forgiveness measure above and beyond the other variables included in the analyses. Specifically, we ran a separate regression model for each of the dimensions of forgiveness (avoidance, revenge, benevolence, and global) for each forgiveness condition. Each regression model included 6 predictor variables: Big Five and the personal component of explanatory style. The results are summarized in Table 6.

**TABLE 6:** Results of regression analyses for the four forgiveness measures for the interpersonal and self-forgiveness conditions (*N* = 160).

Interpersonal forgiveness (*n* = 82)	Avoidance			Revenge			Benevolence			Global		
	**β**	***t***	***p***	**β**	***t***	***p***	**β**	***t***	***p***	**β**	***t***	***p***
Agreeableness	0.03	0.22	0.828	−**0.48**	−**3.67**	**<0.001**	0.14	1.07	0.290	**0.27**	**2.10**	**0.039**
Neuroticism	***0.23***	***1.97***	***0.053***	0.06	0.56	0.579	−0.14	−1.20	0.230	−0.18	−1.62	0.108
Conscientiousness	0.07	0.52	0.599	−0.01	−0.11	0.912	−0.06	−0.51	0.610	−0.07	−0.55	0.586
Openness	−0.10	−0.77	0.442	−0.03	−0.26	0.797	−0.15	−1.22	0.230	0.07	0.56	0.580
Extraversion	−0.06	−0.46	0.648	−0.04	−0.41	0.686	0.17	1.44	0.160	0.00	0.02	0.986
EASQ–S (personal)	0.12	1.12	0.267	0.10	0.97	0.333	0.04	0.38	0.700	−0.09	−0.85	0.400
	*R*^2^ = 0.09			*R*^2^ = 0.25			*R*^2^ = 0.08			*R*^2^ = 0.15		
**Self-forgiveness (*n* = 78)**	**Avoidance**			**Revenge**			**Benevolence**			**Global**		
	**β**	***t***	***p***	**β**	***t***	***p***	**β**	***t***	***p***	**β**	***t***	***p***
Agreeableness	−0.14	−1.27	0.210	**0.26**	**2.07**	**0.042**	0.17	1.35	0.180	−0.12	−1.08	0.285
Neuroticism	***0.20***	***1.88***	***0.065***	***0.20***	***1.69***	***0.096***	−0.02	−0.14	0.890	−**0.33**	−**3.13**	**0.003**
Conscientiousness	−**0.27**	−**2.37**	**0.020**	−0.05	−0.40	0.693	0.09	0.70	0.480	−**0.31**	−**2.69**	**0.009**
Openness	−0.05	−0.52	0.608	0.01	0.07	0.942	−0.09	−0.79	0.430	0.03	0.29	0.772
Extraversion	−0.07	−0.66	0.511	0.15	1.27	0.207	0.09	0.74	0.460	−0.13	−1.27	0.209
EASQ–S (personal)	−**0.27**	−**2.57**	**0.012**	0.07	0.60	0.550	0.03	0.22	0.830	0.01	0.11	0.910
	*R*^2^ = 0.27			*R*^2^ = 0.10			*R*^2^ = 0.07			*R*^2^ = 0.24		

Bolded values indicate *p* < 0.05, whereas bold italics indicate 0.10 > *p* > 0.05. EASQ–S (Personal) = the personal component of the Expanded Attributional Style Questionnaire–Short.

As expected, in the interpersonal forgiveness condition (the top panel), agreeableness and neuroticism remained consistent unique predictors of forgiveness. Specifically, although none of the variables uniquely predicted benevolence motivations, agreeableness uniquely predicted higher levels of global forgiveness and weaker revenge motivations, whereas neuroticism predicted stronger avoidance motivations (albeit marginally at *p* = 0.053).

In the self-forgiveness condition (the bottom panel of Table 6), agreeableness and neuroticism also uniquely predicted forgiveness-related motivations. Specifically, neuroticism predicted lower levels of global forgiveness and stronger (albeit marginally) avoidance motivations. Additionally, higher levels of agreeableness were uniquely associated with stronger revenge motivations toward the self, even though this agreeableness/revenge relationship was only marginally significant at the level of bivariate correlations (*r* = 0.22, *p* = 0.056, Table 5).

More important, conscientiousness remained a unique predictor of self-forgiveness: Higher conscientiousness was uniquely associated with lower levels of avoidance motivations as well as with lower levels of global forgiveness. Similarly, the personal component of explanatory style remained a unique predictor of avoidance motivations. These results corroborate those of the *r*-to-*z* transformation tests reported earlier.

As explanatory analyses, we also ran the same regression models without explanatory style, using only the Big Five factors to predict the forgiveness measures (see Footnote 1 for the rationale for conducting this analysis with only Big Five factors. The results are summarized in Supplementary Table 1 in Supplement B. Given that the personal component of explanatory style was not correlated with any of the Big Five factors (Table 4), it is not surprising that the results remained essentially the same, with two minor differences, both involving neuroticism: With explanatory style no longer in the model, neuroticism significantly predicted avoidance motivations (*p* = 0.044 vs. *p* = 0.065), but its unique predictive power for revenge motivations was no longer even marginally significant (*p* = 0.104 vs. *p* = 0.096).

### 3.3. Secondary analyses: comparison with the HFS

The secondary, more exploratory aim of the study was to compare the current scenario-based assessment of forgiveness with that based on a more prevalently used questionnaire (HFS). Although we did not have any specific predictions, our general expectation was that, whether assessed with transgression scenarios or questionnaires, the interpersonal forgiveness measures should correlate with each other, whereas the self-forgiveness measures should correlate with each other, on the assumption that the two different ways of assessing forgiveness capture, at least in part, the same underlying construct (i.e., dispositional interpersonal or self-forgiveness). The bottom two rows (rows 7 and 8) of Table 5 report how the Other and Self subscales of the HFS correlated with our scenario-based measures of forgiveness.

Consistent with our expectation, the HFS Other subscale correlated with all four scenario-based measures of forgiveness (avoidance, revenge, benevolence, and global) in the interpersonal forgiveness condition. Interestingly, the HFS Self subscale also correlated with three of the four dimensions of interpersonal forgiveness (except for benevolence), although the observed correlations were generally higher for the Other subscale than for the Self subscale.

Contrary to our expectation, however, the HFS Self subscale was not correlated with our scenario-based measures of self-forgiveness, with the exception of avoidance (*r* = –0.30), which was also correlated with the HFS Other subscale equally strongly (*r* = –0.28). These unexpected results seem to suggest that the Self subscale of the HSF is more closely associated with interpersonal forgiveness than with self-forgiveness as assessed with transgression scenarios.

Finally, we conducted multiple regression analyses, in which we used the two HFS subscales (Other and Self) as the respective outcome variables. As summarized in Table 7, the patterns of results for the HFS were substantially different from those for our scenario-based measures, although the differences likely in part reflected sample size differences (*n*’s = 82 and 78 in Table 6 vs. *N* = 160 in Table 7).

**TABLE 7:** Results of regression analyses for the two subscales (other and self) of the Heartland Forgiveness Scale (HFS).

	HFS Other (*N* = 160)			HFS Self (*N* = 160)		
**Measure**	**β**	***t***	***p***	**β**	***t***	***p***
Agreeableness	**0.51**	**6.39**	**<0.001**	**0.16**	**2.18**	**0.031**
Neuroticism	−0.02	−0.27	0.790	−**0.44**	−**6.86**	**<0.001**
Conscientiousness	−0.07	−0.91	0.370	−0.03	−0.47	0.641
Openness	0.07	0.95	0.340	**0.15**	**2.27**	**0.025**
Extraversion	0.01	0.18	0.860	**0.22**	**3.28**	**0.001**
EASQ–S (personal)	0.06	0.89	0.380	0.06	0.91	0.366
	*R*^2^ = 0.27			*R*^2^ = 0.41		

Bolded values indicate *p* < 0.05, whereas italics indicate 0.10 > *p* > 0.05. EASQ–S (Personal) = the personal component of the Expanded Attributional Style Questionnaire–Short.

As shown in Table 7, only agreeableness was a unique predictor of the HFS Other subscale (the left panel). In contrast, four personality dimensions—agreeableness, neuroticism, openness, and extraversion—were unique predictors of the HFS Self subscale (the right panel). Interesting to note, conscientiousness, which was the sole unique Big Five predictor of avoidance motivations and global levels of self-forgiveness for the scenario-based measures (see Table 6), was the only factor that failed to uniquely predict dispositional self-forgiveness as measured with the HFS Self subscale. As shown in Supplementary Table 2 in Supplement B, these regression results remained the same for both forgiveness conditions when explanatory style was excluded from regression models.

## 4. Discussion

The primary goal of the current study was to examine whether the personality traits of forgiving individuals differ for interpersonal and self-forgiveness by directly comparing them using a common set of transgression scenarios. By applying the motivational-change view of forgiveness (McCullough and Hoyt, 2002; Hall and Fincham, 2005) to both types of forgiveness and assessing the same transgression-related motivations, the current study provided a direct comparison of the personality correlates of interpersonal versus self-forgiveness. The secondary goal was to compare the current scenario-based assessment of dispositional forgiveness with the more decontextualized assessment of forgiveness based on general statements (i.e., HFS Self and Other subscales). Below, we will summarize the main findings and discuss their implications.

### 4.1. Aim 1: personality correlates of dispositional interpersonal and self-forgiveness

#### 4.1.1. Similarities and differences in the personality correlates of two types of forgiveness

Regarding the first aim, the primary finding was that the personality traits of individuals who are inclined to forgive others (interpersonal forgiveness) and those who are inclined to forgive themselves (self-forgiveness) show some similarities as well as differences, when assessed with scenarios. Starting with similarities, neuroticism and agreeableness were unique predictors of not only interpersonal forgiveness but also self-forgiveness (especially, agreeableness for revenge motivations and neuroticism for avoidance motivations).

Our results showed that individuals who tend to be emotionally sensitive and thin-skinned (i.e., high in neuroticism) may experience greater difficulty in facing the other party, regardless of whether they are the victims or the transgressors, because such a confrontation would no doubt invoke negative emotions with which they might not be able to cope well. Such emotional difficulty in turn may lead to maladaptive ruminative thinking, which makes it even more difficult to promote any type of forgiveness.

Another underlying commonality concerned the role of agreeableness. Regression results showed that agreeableness uniquely predicted revenge motivations for both interpersonal and self-forgiveness. As expected, individuals who tend to be kind and sympathetic to other people (i.e., high in agreeableness) are unlikely to develop and harbor strong revenge motivations toward the transgressor when they were the victim of a transgression (interpersonal forgiveness). Interestingly, those same individuals high in agreeableness may not be as kind and as sympathetic toward themselves when they are the transgressor. As shown in the regression results for revenge motivations in Table 6, they seem to harbor stronger revenge or self-punishing motivations toward themselves (i.e., feeling more strongly that they should pay the price), perhaps because they might feel particularly sympathetic toward the victim.

Our results also demonstrated that self-forgiveness, as assessed with the current scenario-based approach, implicates some additional unique personality traits that are not implicated in interpersonal forgiveness. First, as hypothesized, conscientiousness uniquely predicted self-forgiveness, but not interpersonal forgiveness, for global forgiveness and avoidance motivations. This unique role of conscientiousness in self-forgiveness suggests that individuals who tend to pride themselves on their dependability and dutifulness (i.e., high in conscientiousness) may have a particularly difficult time forgiving themselves when they believe that their temporary negligence or carelessness led to the disastrous outcome. Such trait characteristics associated with conscientiousness may not matter as much if they are the victims of the transgression, because the disastrous outcome was not caused by their own negligence or carelessness.

Moreover, also as hypothesized, one’s tendency to internalize failure (the personal component of explanatory style) was a unique predictor of avoidance motivations in the self-forgiveness condition only. Considering the low reliability of this measure noted earlier, this finding must be interpreted with caution, but it nonetheless suggests the potential usefulness of explanatory style in future studies on the personality correlates of self-forgiveness.

#### 4.1.2. Different patterns of correlations among three transgression-related motivations

As briefly noted earlier (section “3.1.2. Forgiveness measures: bivariate correlations”), one curious finding that points to another potential difference between the two types of forgiveness is that, whereas the three transgression-related motivations (avoidance, revenge, and benevolence) correlated substantially with each other in the interpersonal forgiveness condition, avoidance and revenge motivations did not correlate with each other (*r* = 0.02) in the self-forgiveness condition (see Table 3). Although speculative, at least two (not necessarily mutually exclusive) explanations seem possible.

First, this correlational dissociation observed in the self-forgiveness condition can be interpreted from the perspective of the conception of self-forgiveness mentioned earlier (section “1.1. Motivational-change framework for interpersonal and self-forgiveness”) that includes two key components: (a) accepting responsibility for wrongdoing (related to avoidance and benevolence motivations) and (b) restoring one’s sense of self (related to revenge motivation). From this perspective, one might expect a substantial correlation between avoidance and benevolence motivations (two facets of accepting responsibility), but a weaker one between avoidance/benevolence motivations and revenge motivations (facets of accepting responsibility vs. self-restoration). The observed correlations in the self-forgiveness condition are consistent with this explanation.

Second, more mechanistically, another possibility is that high levels of revenge or self-punishing motivations (i.e., feeling that “I should pay”) trigger two different reactions among the transgressors. Some individuals may actively try to approach the victim and seek amends to restore their relationship, whereas other individuals may be paralyzed by strong negative emotions (e.g., guilt and shame) and hence actively try to avoid the victim (e.g., too ashamed to face the victim). If equivalent levels of feeling “I should pay” can elicit two such opposing reactions among different individuals, then that may lead to the observed lack of correlations between revenge and avoidance motivations in the self-forgiveness condition.

### 4.2. Aim 2: comparison with forgiveness assessed by the HFS subscales

A more exploratory aim of the study was to compare our scenario-based assessment of forgiveness to a more prevalent questionnaire-based assessment, which typically involves self-ratings on decontextualized statements, such as *I hold grudges against myself for negative things I’ve done*. To the best of our knowledge, this is the first study in which two types of forgiveness (interpersonal and self-forgiveness) were both assessed with multiple methods (e.g., scenarios and questionnaires). As such, our results provide new insights into the similarities and differences between two methods of assessing forgiveness.

Based on the assumption that scenarios-based and questionnaire-based assessments capture the same underlying construct, at least to some extent, our general expectation was that the interpersonal forgiveness measures would correlate with each other, whereas the self-forgiveness measures would correlate with each other. As shown at the bottom of Table 5 (rows 7 & 8), this expectation of ours was consistent with the results from the interpersonal forgiveness condition, but not with the results from the self-forgiveness condition.

As expected, the HSF other subscale was consistently correlated with all four dimensions of interpersonal forgiveness assessed with transgression scenarios (see row 7 of Table 5). Moreover, except for avoidance, the Other subscale was not correlated with the other three dimensions of self-forgiveness assessed with scenarios. Thus, the two methods of assessment showed some convergence for interpersonal forgiveness.

In contrast, the results obtained for self-forgiveness did not show the expected pattern. In fact, as shown in row 8 of Table 5, the Self subscale of the HFS was more consistently related to the scenario-based measures of *interpersonal* forgiveness (correlated with avoidance, revenge, and global measures) than to the scenario-based measures of *self*-forgiveness (correlated only with avoidance). These results suggest that, even though the HFS Self subscale has been a useful tool for assessing dispositional self-forgiveness, it may not be as successful in predicting levels of self-forgiveness in more specific, contextualized situations. Thus, some caution is necessary in generalizing the results based on the HFS Self subscale to other situations involving, or other methods of assessing, self-forgiveness.

Another interesting difference between the two methods of assessing dispositional self-forgiveness can be seen in the regression results in Tables 6, 7. In our scenario-based assessment, conscientiousness was a unique predictor of self-forgiveness (specifically for avoidance motivations and global forgiveness), but that was not the case for the HFS Self subscale (Table 6). In fact, the other four Big Five dimensions (agreeableness, neuroticism, extraversion, and, to a lesser extent, openness) were stronger predictors of the HFS Self subscale than was conscientiousness (Table 7). This difference may have to do with the fact that the cause of the tragedy (one’s temporary negligence) was clearly specified in the transgression scenarios used in the current study, whereas such causal attributions are difficult to make in general statements like those used in the HFS Self subscale (e.g., *I hold grudges against myself for negative things I’ve done*). Thus, the relevance of conscientiousness as a predictor of self-forgiveness may depend on the extent to which one can unambiguously identify the transgression’s likely cause.

More generally, these non-convergent results from scenario-based and questionnaire-based methods observed in the self-forgiveness condition point to the importance of using multiple assessment methods within a single study in future research. We argue that a scenario-based assessment—an approach underused in self-forgiveness research—could serve as a useful tool in this regard.

As we demonstrated in the current study, one advantage of the scenario-based assessment of forgiveness is that it allows researchers to directly compare interpersonal and self-forgiveness using the same set of transgression scenarios. Such direct comparisons would be difficult with an approach based on general decontextualized statements in questionnaires or participants’ recollections of actual transgression-related episodes. Another advantage of the scenario-based approach is that it also allows researchers to manipulate the specifics of the transgression-related events described in the scenarios (e.g., the presence vs. absence of apologies or an advance warning from the future victim) and compare the impacts of such changes on forgiveness ratings. In fact, Leunissen et al. (2013) did just that and uncovered interesting asymmetries in the roles of apologies in interpersonal forgiveness versus self-forgiveness (a victim’s need for apologizes vs. a transgressor’s willingness to apologize). Given that it is impossible—let alone ethically inappropriate—to experimentally manipulate serious transgressions of the sort described in our scenarios in everyday settings, the scenario-based approach should be able to play a unique role in forgiveness research.

Of course, the scenario-based approach has its own limitations and biases (e.g., Fehr et al., 2010, for further discussion). For example, even when realistic and relatable scenarios are used, they are still based on imagined events and thus lack the realism of actual, personally experienced transgressions. Perhaps for this reason, in their meta-analytic review, Fehr et al. (2010) observed that scenario-based methods of assessing forgiveness have stronger effects on cognitions (e.g., ratings on intent, responsibility) than do recall-based methods, whereas the latter has stronger effects on affect (e.g., ratings on sympathy, negative moods), thus pointing to the complementary nature of these different methods of assessing forgiveness. Such findings further reinforce the need for multiple assessment approaches (scenario-based, questionnaire-based, recall-based, etc.) in future investigations on the personality correlates of forgiveness.

### 4.3. Limitations of the study and future directions

Although the current study has several notable strengths, we acknowledge here some limitations of the study and suggest possible future research directions. First, because we used a between-subjects design and created separate groups for interpersonal and self-forgiveness (primarily to avoid any carry-over effects), the resulting sample size in each group is not large (*n*’s = 82 and 78, respectively). Additionally, the internal reliability for the personal component of our explanatory style measure was low, despite showing a predicted association with some facets of self-forgiveness. Thus, some caution is needed in interpreting the results, and we advocate for replications with larger samples and better assessments of explanatory style.

Second, we used only 6 scenarios for the assessment of dispositional forgiveness in this study. Although this number is comparable to that in Berry et al.’s (2001) TNTF scale (5 scenarios), it is not as high as McCullough and Hoyt (2002) recommended—“perhaps as many as 12–16” (p. 1570)—if the goal is to systematically vary the scenarios along multiple dimensions to represent a broad range of transgression characteristics and situations. In this study, however, we made our scenarios similar in structure to reliably capture stable individual differences in forgiveness even with a relatively small number of scenarios. Judging from the high internal reliability estimates observed for all of our forgiveness measures (in the 0.72–0.93 range; see Table 2), we were successful in this regard, but future studies should use a larger number of scenarios.

Third, although this relative homogeneity of the scenarios was intentionally introduced, it necessarily limited the generalizability of the current results to other types of transgression characteristics (e.g., less severe transgressions) and victim–transgressor relationships (e.g., mere acquaintances, spouces and close family members). Given that this was the first study in which the personality correlates of interpersonal and self-forgiveness were directly compared using the same set of realistic scenarios, this tradeoff may be justified. As suggested earlier, however, one advantage of the scenario-based approach lies in the relative ease of systematically varying various parameters within transgression scenarios. Thus, future studies should explore the impacts of such parametric changes on readers’ forgiveness-related motivations and their associations with different personality variables.

Fourth, the current study was based on the conception of interpersonal forgiveness derived from McCullough et al.’s (1998) motivational-change view and its adaptation to self-forgiveness, but, as noted earlier (section “1.1. Motivational-change framework for interpersonal and self-forgiveness”), we made one key change to Hall and Fincham (2005) conceptualization of self-forgiveness. Specifically, we intentionally made the victim (the other party), rather than the transgressor (the self), the target of benevolent actions to capture the important role of seeking reconciliation and restoring the relationship with the victim in fostering self-forgiveness. We acknowledge, however, that it would have been equally justifiable to keep Hall and Fincham’s (2005) original conceptualization of benevolence motivations and designate the self as the target of benevolent actions, because self-forgiveness clearly must go beyond reconciliation and also involve making peace with one’s action and being kinder to oneself. Thus, comparing the personality correlates of these two different operationalizations of benevolent motivations in self-forgiveness would be a worthwhile endeavor in future research.

More generally, as also noted earlier (section “1.1. Motivational-change framework for interpersonal and self-forgiveness”), there are newer and broader-scope conceptions of forgiveness, especially for self-forgiveness (e.g., Cornish et al., 2018; Worthington, 2020; Woodyatt and Wenzel, 2020). For example, Webb et al.’s (2017) proposed definition includes some additional components unique to self-forgiveness, such as accepting personal responsibility, human connectedness, and efforts for personal growth and change. Assessing such unique dimensions of self-forgiveness and examining their personality correlates would also be an important future research direction.

Fifth, in self-forgiveness research, one measurement challenge has been to differentiate genuine forms of self-forgiveness from other not-so-genuine forms, such as deflecting blames and not feeling self-punitive desires toward the transgression at the onset (e.g., Woodyatt and Wenzel, 2013). Given that the goal of the study was to compare the personality correlates of dispositional interpersonal and self-forgiveness, we did not directly attempt to address this important measurement issue in this study. In future research, it would be interesting to examine how the personality correlates of genuine self-forgiveness would be different from those of its not-so-genuine counterparts, such as self-exoneration (i.e., high overall self-forgiveness but low revenge or self-punishing motivations; Cornish et al., 2018).

Sixth, by using the BFI, we assessed the Big Five factors at a general level, without considering different subdimensions identified for each factor. As suggested by Ross et al. (2004) and Mullet et al. (2005), different subdimensions of a particular personality factor (such as those assessed in NEO-PI) are likely to be differentially associated with dispositional interpersonal or self-forgiveness. For example, in our account, some specific attributes of conscientiousness like “dependability” and “dutifulness” were emphasized, but some other attributes of conscientiousness like “productive” or “achievement striving” may not be as strongly related to self-forgiveness. Future research needs to specify the personality correlates of interpersonal and self-forgiveness at a more micro level of analysis.

Finally, the existing research on the personality correlates of forgiveness has been dominated by the Big Five model of personality (McCrae and John, 1992). Although further work is needed to obtain a more consistent picture of the Big Five correlates of dispositional self-forgiveness across different assessment methods, there already exists substantial converging evidence for the Big Five correlates for interpersonal forgiveness (primarily, agreeableness and neuroticism). Thus, it is important to explore other personality correlates of forgiveness to achieve a more comprehensive understanding of the characteristics of forgiving individuals. Some promising personality and other psychological traits that have already been studied in forgiveness research include (but are not limited to): explanatory style (current study), self-compassion (e.g., Oral and Arslan, 2017), self-criticism (e.g., Barcaccia et al., 2020), and ruminative tendencies (e.g., McCullough et al., 1998; Oral and Arslan, 2017). Of course, these and other forgiveness-related psychological traits have been examined before, but only sporadically, especially when compared to the Big Five factors. Thus, more systematic investigations of such conceptually more specific personality correlates should contribute substantially to a more comprehensive understanding of what makes some individuals more forgiving than others.

## 5. Conclusion

Despite these limitations, the direct comparison between interpersonal and self-forgiveness attempted in the current study shed new light on their similarities and differences in the personality correlates for the two types of forgiveness. Moreover, the comparison of two assessment methods also highlighted the importance of using multiple methods in examining interpersonal and self-forgiveness. We argue that systematically elucidating similarities and differences between interpersonal and self-forgiveness may be helpful in future attempts to develop a unified model that can encompass both types of forgiveness within a single theoretical framework. In particular, identifying similarities and differences in the personality traits of individuals inclined to forgive others versus forgive themselves—as was done here—will likely provide an important foundation for such theoretical development.

## Data availability statement

The datasets presented in this study can be found in online repositories. The names of the repository/repositories and accession number(s) can be found below: https://osf.io/wbzs3/ (Open Science Framework).

## Ethics statement

The studies involving humans were approved by the Internal Review Board University of Colorado Boulder. The studies were conducted in accordance with the local legislation and institutional requirements. The participants provided their written informed consent to participate in this study.

## Author contributions

LH and AM initially conceptualized and design the study, developed the stimulus materials, and wrote a preliminary version of the manuscript. LH collected the data with some help from NC and JL. LH initially conducted the data analysis with some guidance from AM and JL. NC conducted further data analyses and prepared the final version of the datasets to be shared on the project site at Open Science Framework. AM took the lead in revising the manuscript. LH, NC, and JL also contributed to the finalization of the manuscript. All authors contributed to the article and approved the submitted version.

## Footnotes

1 According to a recent meta-analysis (Credé et al., 2017), grit is strongly correlated with conscientiousness (*r* = 0.66, *k* = 22; see also Ponnock et al., 2020). Similarly, ruminative tendencies (anger or depressive) have been known to be substantially associated with neuroticism (e.g., du Pont et al., 2019). Therefore, the results from these studies (Oral and Arslan, 2017; Walker, 2017) might look different if the non-Big Five variables were excluded from their respective regression analyses.

2
https://osf.io/wbzs3/

3 One “catch” question asked participants to select “strongly agree” as the response, whereas the other asked them to select “strongly disagree.” Failure to respond correctly to both of these direct requests was interpreted as indicative of random or inattentive responding (Maniaci and Rogge, 2014).

4 The interpersonal forgiveness condition (*n* = 82) included 40 women and 42 men, whereas the self-forgiveness condition (*n* = 78) included 44 women and 34 men. The participants’ age ranged from 18 to 29 years old, although most of them were in the 18–20 range. The mean age was 18.81 years old (*SD* = 1.18) in the interpersonal personal condition and 19.13 years old (*SD* = 2.40) in the self-forgiveness condition. Finally, the race/ethnicity breakdown of the participants in the two conditions (interpersonal vs. self-forgiveness) were: (a) White 72% vs. 83%, (b) Asian 12% vs. 9%; (c) African-American 2% vs. 0%; (d) Hispanic 9% vs. 6%; and (e) Other (including multiracial) 5% vs. 1%. These proportions are comparable to those of undergraduate students at the University of Colorado Boulder.

5 Although these measures are not discussed in this article, their data are available in the publicly shared data (https://osf.io/wbzs3/). The dependent measure for both tasks was the total number of target items correctly remembered in the correct serial position.

6 To avoid extreme responses (e.g., 2 min, 1 million years), we used a nominal scale for this item: 1 = within one day; 2 = within 1 week; 3 = within 1 month; 4 = within 3 months; 5 = within 1 year; 6 = within 3 years; 7 = within 10 years; and 8 = never. Because this measure and the other measure of global forgiveness (Eventual) correlated substantially in both the interpersonal and self-forgiveness conditions (*r* = 0.63 and.66, respectively), we treated the Time scale as though it were an ordinal scale and aggregated the two global forgiveness measures by computing the *z*-score averages.

7 Our exploratory analysis revealed that one of the 9 failure prompts used in our study was negatively correlated with the total score, but excluding the ratings associated with this prompt did not substantially improve the measure’s internal reliability (α = 0.32). Using 9 of the failure prompts (instead of all 12) due to time constraints also likely contributed to lower-than-expected internal reliability estimate.

8 Although we collapsed the data for the two forgiveness conditions in Table 4, there were no significant group differences for any of these measures, suggesting that the two groups of participants were equivalent before reading the transgression narratives.
