# Supplementary material for: Personality correlates of dispositional forgiveness: a direct comparison of interpersonal and self-forgiveness using common transgression scenarios
Source: Front Psychol. 2023 Nov 1;14:1218663. doi: 10.3389/fpsyg.2023.1218663 (PMC10646512; doi:10.3389/fpsyg.2023.1218663)
Supplement: Supplementary file 1 [file Data_Sheet_1.PDF]

Supplement A
Fictional Scenarios Used for the Study

Following each scenario is a 10-item questionnaire assessing *Offense Severity* (1 item), *Recent Experience* (1 item), *Avoidance* (2 items), *Revenge* (2 items), *Benevolence* (2 items), and *Global Forgiveness* (2 items, consisting of *Eventual* forgiveness and forgiveness over *Time*).

As shown below, the background items—Offense Severity and Recent Experience—were always presented at the beginning in that order. The next six items representing three transgression-related motivations (avoidance, revenge, and benevolence) were randomized once for each scenario (with the constraint that the two items representing the same motivation could not appear consecutively) and presented in the same fixed order to all participants. The last two items were about global levels of forgiveness for all scenarios, with the eventual forgiveness measure always appearing before the final time measure.

Offense Severity was rated on a 5-point Likert scale, ranging from "not upset at all" (1) to "extremely upset" (5). The items for Recent Experience, Avoidance, Revenge, Benevolence, and Eventual forgiveness were each rated a 6-point Likert scale ranging from "strongly disagree" (1) to "strongly agree" (6). Forgiveness over Time was rated on an 8-point nominal scale ranging from "one day" (1) to "never" (8) (see Footnote 5 in the main text for more information about this scale). Note that the two items assessing the levels of Global Forgiveness, Eventual and Time, were both reverse coded (denoted "R" below) so that higher scores would indicate higher levels of forgiveness.

Scenario 1 (House Fire)

Interpersonal Forgiveness

You have recently moved into a new house, and you decide to throw a housewarming party. You invite several of your friends, order catered food, and provide drinks. When everyone gets to the party, you give the tour of the new apartment and show everyone the food and drinks.

As things progress, you notice one of your friends smoking a cigarette on your patio, and—once he is finished—he casually throws it into the yard. You approach him and give him a friendly reminder to use the ashtray, for fire danger is very high at the moment, and your neighbor's house recently burnt to the ground because of a careless smoker. He says that he understands.

Towards the end of the night you see your friend once again absentmindedly throw a cigarette into the yard. However, this time, you see that it hits some dry leaves. Next thing you know the fire alarms go off and the party scatters outside. Your house completely burns down.

1. How upset would you be if this happened to you? (Offense Severity)
2. I recently experienced a similar situation. (Recent Experience)
3. I would be trying to keep as much distance between us as possible. (Avoidance)
4. Despite what he did, I would be working hard to bury the hatchet and move forward with our relationship. (Benevolence)
5. I would want to make him pay. (Revenge)
6. I would be striving to achieve reconciliation. (Benevolence)
7. I would be avoiding him. (Avoidance)
8. I would want him to get what he deserves. (Revenge)
9. I would never be able to forgive him. (Eventual: R)

10. How long would it take you to forgive him (please select the response that aligns closest with your thoughts/opinions)? (Time: R)

Self-Forgiveness

Your friend has recently moved into a new house and is throwing a housewarming party. He invited several of your friends, ordered catered food, and provided drinks. When everyone gets to the party, he gives a tour of the new apartment and shows everyone the food and drinks.

As things progress, you decide to smoke a cigarette on the patio, and—once you are finished—you casually throw it into the yard. Your friend approaches you and gives you a friendly reminder to use the ashtray, for the fire danger is very high at the moment, and his neighbor's house recently burnt to the ground because of a careless smoker. You say that you understand.

Towards the end of the night you once again absentmindedly throw a cigarette into the yard. However, this time, you see that it hits some dry leaves. Next thing you know the fire alarms go off and the party scatters outside. The house completely burns down.

1. How upset would you be if you did this? (Offense Severity)
2. I recently experienced a similar situation. (Recent Experience)
3. I would be trying to keep as much distance between us as possible. (Avoidance)
4. Despite what I did, I would be working hard to bury the hatchet and move forward with our relationship. (Benevolence)
5. I should have to pay for what I did. (Revenge)
6. I would be striving to achieve reconciliation. (Benevolence)
7. I would be avoiding him. (Avoidance)
8. I should get what I deserve. (Revenge)
9. I would never be able to forgive myself. (Eventual: R)
10. How long would it take you to forgive yourself (please select the response that aligns closest with your thoughts/opinions)? (Time: R)

Scenario 2 (Pet Death)

Interpersonal Forgiveness

You have been saving up your vacation days for the entire year, and you finally decide to take two weeks and go on your dream vacation. The only problem is that you have a dog and cannot leave it alone for so long. Putting your dog in a kennel for two weeks is not only too expensive, but you care about your dog too much to leave it in such a place.

One of your friends offers to watch your dog. She works from home so the dog will get plenty of attention. You are very relieved that this problem is resolved, and you proceed to take your dream vacation. Before you go, you remind your friend to make sure not to leave any doors open, for your dog likes to get outside whenever it can.

Your vacation goes very well. The day you come to pick up your dog, your friend goes to the grocery store and accidentally leaves the front door ajar. You come to pick up the dog before your friend gets back, and you find it on the road; it was hit by a car.

1. How upset would you be if this happened to you? (Offense Severity)
2. I recently experienced a similar situation. (Recent Experience)
3. I would be trying to keep as much distance between us as possible. (Avoidance)

4. Despite what she did, I would be working hard to bury the hatchet and move forward with our relationship. (Benevolence)
5. I would want to make her pay. (Revenge)
6. I would be striving to achieve reconciliation. (Benevolence)
7. I would be avoiding her. (Avoidance)
8. I would want her to get what she deserves. (Revenge)
9. I would never be able to forgive her. (Eventual: R)
10. How long would it take you to forgive her (please select the response that aligns closest with your thoughts/opinions)? (Time: R)

Self-Forgiveness

Your friend has been saving up her vacation days for the entire year, and she finally decides to take two weeks and go on her dream vacation. The only problem is that she has a dog and cannot leave it alone for so long. Putting her dog in a kennel for two weeks is not only too expensive, but she cares about the dog too much to leave it in such a place.

You offer to watch the dog. You work from home, so the dog will get plenty of attention. Your friend is very relieved that this problem is resolved, and she proceeds to take her dream vacation. Before she goes, she reminds you to make sure not to leave any doors open, for her dog likes to get outside whenever it can.

Your friend's vacation goes very well. The day she comes to pick up the dog, you go to the grocery store and leave the front door ajar. Your friend comes to pick up the dog before you get back, and she finds it on the road; it was hit by a car.

1. How upset would you be if you did this? (Offense Severity)
2. I recently experienced a similar situation. (Recent Experience)
3. I would be trying to keep as much distance between us as possible. (Avoidance)
4. Despite what I did, I would be working hard to bury the hatchet and move forward with our relationship. (Benevolence)
5. I should have to pay for what I did. (Revenge)
6. I would be striving to achieve reconciliation. (Benevolence)
7. I would be avoiding her. (Avoidance)
8. I should get what I deserve. (Revenge)
9. I would never be able to forgive myself. (Eventual: R)
10. How long would it take you to forgive yourself (please select the response that aligns closest with your thoughts/opinions)? (Time: R)

Scenario 3 (Group Project)

Interpersonal Forgiveness

In one of your upper division degree requirement courses, you are assigned a group project. You are placed in a group with one other classmate, and you and he are assigned a project topic. There are 100 points in the entire class, and this project is worth thirty points, 10 being an individual grade and the other 20 being a group grade.

You have known your groupmate for several years, and you two have moved through the degree path in a similar manner. Your group meets to discuss the project, and you and he agree that this project would be best handled by dividing the project into two equal sections: you will summarize the

project goals, introduce the topic, and present the problem solving process, whereas he will analyze the data, summarize the findings, and present a conclusion.

The project takes several weeks, but on the presentation day you are well prepared, but your groupmate is not because he has procrastinated for too long. You two had previously decided to meet half an hour before class to merge the sections together, but he does not show up. The class begins, and he has still not arrived. Your missing groupmate decides that you are better off without him, and so he never shows up for class.

Your professor is unsympathetic with your group, and even though you received a 9/10 on the individual grade, your group grade was 10/20; so you ended up with a 63%, which severely affected your grade in the course overall and lowered your GPA substantially. You are now sure that you cannot keep receiving your scholarships next year.

1. How upset would you be if this happened to you? (Offense Severity)
2. I recently experienced a similar situation. (Recent Experience)
3. I would be trying to keep as much distance between us as possible. (Avoidance)
4. Despite what he did, I would be working hard to bury the hatchet and move forward with our relationship. (Benevolence)
5. I would want to make him pay. (Revenge)
6. I would be striving to achieve reconciliation. (Benevolence)
7. I would be avoiding him. (Avoidance)
8. I would want him to get what he deserves. (Revenge)
9. I would never be able to forgive him. (Eventual: R)
10. How long would it take you to forgive him (please select the response that aligns closest with your thoughts/opinions)? (Time: R)

Self-Forgiveness

In one of your upper division degree requirement courses, you are assigned a group project. You are placed in a group with one other classmate, and you and he are assigned a project topic. There are 100 points in the entire class, and this project is worth thirty points, 10 being an individual grade and the other 20 being a group grade.

You have known your groupmate for several years, and you two have moved through the degree path in a similar manner. Your group meets to discuss the project, and you and he agree that this project would be best handled by dividing the project into three equal sections: you will summarize the project goals, introduce the topic, and present the problem solving process, whereas he will analyze the data, summarize the findings, and present a conclusion.

The project takes several weeks, but on the presentation day you are not at all prepared because you have procrastinated for too long, even though your groupmate is well prepared. You two had previously decided to meet half an hour before class to merge the sections together, but you do not show up. The class eventually begins without you. You decide that your groupmate is better off without you, and so you never show up for class.

Your professor is unsympathetic with your group, and even though your groupmate received a 9/10 on his individual grade, the group grade was 10/20; so he ended up with a 63%, which severely affected his grades in the course overall and lowered his GPA substantially. He is now sure that he cannot keep receiving his scholarships next year.

1. How upset would you be if you did this? (Offense Severity)
2. I recently experienced a similar situation. (Recent Experience)
3. I would be trying to keep as much distance between us as possible. (Avoidance)

4. Despite what I did, I would be working hard to bury the hatchet and move forward with our relationship. (Benevolence)
5. I should have to pay for what I did. (Revenge)
6. I would be striving to achieve reconciliation. (Benevolence)
7. I would be avoiding him. (Avoidance)
8. I should get what I deserve. (Revenge)
9. I would never be able to forgive myself. (Eventual: R)
10. How long would it take you to forgive yourself (please select the response that aligns closest with your thoughts/opinions)? (Time: R)

Scenario 4 (Employment Opportunity)

Interpersonal Forgiveness

It is the end of the fall semester, and you are about to graduate from college. You have been sending out resumes for months now hoping to transition directly into a career, and you have just been asked to interview for your dream job. The job is in Chicago, where a friend of yours lives.

The interview is several days before Christmas, and so when you try to book the flight, you see that almost every day is fully booked. Your only option is to arrive on the day of the interview, and the earliest available flight arrives only four hours before the interview. You book the flight, but since the window of time is so narrow, and you don't want to risk having to rely on a cab, you ask your friend in Chicago if she can pick you up. She says that she will happily pick you up and take you to the interview. Before you get off the phone with her, you explain the very short window of time and the necessity that she be there on time. She reassures you that she will be there on time.

Your flight ends up arriving early. You decide to call your friend to let her know, but she doesn't respond. Thirty more minutes pass, and you call her again, but again she doesn't answer. After waiting for an hour, you look around for a cab, but due to the holidays, there are many, many people waiting in line, and the line is not moving at all. You call the cab company, but they don't have an available driver for another three hours.

You eventually get hold of your friend. She explains that she forgot about your flight and saw a movie with another friend. While in the theater, she could not hear her phone. You end up missing your interview, and when you call the company to reschedule, they let you know that the position has already been filled.

1. How upset would you be if this happened to you? (Offense Severity)
2. I recently experienced a similar situation. (Recent Experience)
3. I would be trying to keep as much distance between us as possible. (Avoidance)
4. Despite what she did, I would be working hard to bury the hatchet and move forward with our relationship. (Benevolence)
5. I would want to make her pay. (Revenge)
6. I would be striving to achieve reconciliation. (Benevolence)
7. I would be avoiding her. (Avoidance)
8. I would want her to get what she deserves. (Revenge)
9. I would never be able to forgive her. (Eventual: R)
10. How long would it take you to forgive her (please select the response that aligns closest with your thoughts/opinions)? (Time: R)

Self-Forgiveness

It is the end of the fall semester, and your friend is about to graduate from college. She has been sending out resumes for months now hoping to transition directly into a career, and she has just been asked to interview for her dream job. The job is in Chicago, where you live.

The interview is several days before Christmas, and so when she tries to book the flight, she sees that almost every day is fully booked. Her only option is to arrive on the day of the interview, and the earliest available flight arrives only four hours before the interview. She books the flight, but since the window of time is so narrow, and she doesn't want to risk having to rely on a cab, she asks if you can pick her up. You say that you will happily pick her up and take her to the interview. Before she gets off the phone with you, she explains the very short window of time and the necessity that you be there on time. You reassure her that you will be there on time.

On the day of your friend's arrival, you are invited to see an afternoon movie. You—forgetting that you have to pick up your friend—agree to go see the movie. When the movie is over, you discover several voicemails on your phone. She decided to call you when she arrived, but your phone was silenced in the movie theater. After waiting for an hour, your friend looked around for a cab, but due to the holidays, there were many, many people waiting in line, and the line was not moving at all. She called the cab company, but they didn't have an available driver for another three hours.

You eventually get hold of your friend. You explain that you forgot about her flight and saw a movie with another friend. While in the theater, you could not hear your phone. She ended up missing her interview, and when she called the company to reschedule, they let her know that the position had already been filled.

1. How upset would you be if you did this? (Offense Severity)
2. I recently experienced a similar situation. (Recent Experience)
3. I would be trying to keep as much distance between us as possible. (Avoidance)
4. Despite what I did, I would be working hard to bury the hatchet and move forward with our relationship. (Benevolence)
5. I should have to pay for what I did. (Revenge)
6. I would be striving to achieve reconciliation. (Benevolence)
7. I would be avoiding her. (Avoidance)
8. I should get what I deserve. (Revenge)
9. I would never be able to forgive myself. (Eventual: R)
10. How long would it take you to forgive yourself (please select the response that aligns closest with your thoughts/opinions)? (Time: R)

Scenario 5 (Moving Help)

Interpersonal Forgiveness

The lease for your apartment ends in several weeks. You have already found a new apartment in an ideal area. The lease is signed, but you can't start moving until just two days before the lease at your old apartment ends, and property management has told you that the new tenants are moving in the day after your lease ends.

You don't have too many things to move, but there are several large items—namely, a couch, a bed, two bookshelves, and a dining room table—that require more than one person to move. Your friend offers to help you move, but he is only free on the day before you are supposed to be moved out. You decide to rent a storage truck for that day, and hopefully—between the two of you—you can tackle all of the heavy furniture.

The day begins and your friend calls you. He lets you know that he was invited to a tennis match in the early morning, but that it should be over well before you are scheduled to pick up the moving truck. You remind him that this is your only day to move the big furniture, and he reassures you that he will be there.

Your friend's tennis match tied and went into extra sets. The match was so exciting that he decided to stay until the match was over. As a result, you weren't able to move even half of the furniture, and the new tenants arrive at your old apartment tomorrow morning.

1. How upset would you be if this happened to you? (Offense Severity)
2. I recently experienced a similar situation. (Recent Experience)
3. I would be trying to keep as much distance between us as possible. (Avoidance)
4. Despite what he did, I would be working hard to bury the hatchet and move forward with our relationship. (Benevolence)
5. I would want to make him pay. (Revenge)
6. I would be striving to achieve reconciliation. (Benevolence)
7. I would be avoiding him. (Avoidance)
8. I would want him to get what he deserves. (Revenge)
9. I would never be able to forgive him. (Eventual: R)
10. How long would it take you to forgive him (please select the response that aligns closest with your thoughts/opinions)? (Time: R)

Self-Forgiveness

The lease for your friend's apartment ends in several weeks. He has already found a new apartment in an ideal area. The lease is signed, but he can't start moving until just two days before the lease at his old apartment ends, and property management has told him that the new tenants are moving in the day after his lease ends.

He doesn't have too many things to move, but there are several large items—namely, a couch, a bed, two bookshelves, and a dining room table—that require more than one person to move. You offer to help him move, but you are only free on the day before he is supposed to be moved out. He decides to rent a storage truck for that day, and hopefully—between the two of you—you can tackle all of the heavy furniture.

The day begins and you get a call. Some other friends of yours are playing tennis in the early morning, but it should be over well before your friend is scheduled to pick up the moving truck. You call the friend whom you told that you would help move and let him know. He reminds you that this is his only day to move the big furniture, and you reassure him that you will be there.

Your tennis match tied and went into extra sets. The match was so exciting that you decided to stay until the match was over. As a result, your friend wasn't able to move even half of the furniture, and the new tenants arrive at his old apartment tomorrow morning.

1. How upset would you be if you did this? (Offense Severity)
2. I recently experienced a similar situation. (Recent Experience)
3. I would be trying to keep as much distance between us as possible. (Avoidance)
4. Despite what I did, I would be working hard to bury the hatchet and move forward with our relationship. (Benevolence)
5. I should have to pay for what I did. (Revenge)
6. I would be striving to achieve reconciliation. (Benevolence)
7. I would be avoiding him. (Avoidance)

8. I should get what I deserve. (Revenge)
9. I would never be able to forgive myself. (Eventual: R)
10. How long would it take you to forgive yourself (please select the response that aligns closest with your thoughts/opinions)? (Time: R)

Scenario 6 (Car Theft)

Interpersonal Forgiveness

You have been living in the same apartment for quite some time, and you just got a new roommate. Everything is going well with you two, but she recently got a new job, and the job requires that she has reliable transportation. Given that you work only several blocks from your house, you offer to let her use your car.

One day when you leave the house to run errands, you notice that your car had been left unlocked. You confront your roommate about it; she apologizes, and says that it won't happen again.

Since then you hadn't been paying attention as to whether your roommate had or had not been making sure to lock your car at night, but, on the news, you hear that there has been a rise in car burglaries in your neighborhood. Just to be cautious, you inform your roommate and once again remind her to be vigilant about locking the car.

The very next morning you awake and see that your car has been broken into and stripped of all electronics and valuable parts. Your roommate had used the car last night and forgot to lock it.

1. How upset would you be if this happened to you? (Offense Severity)
2. I recently experienced a similar situation. (Recent Experience)
3. I would be trying to keep as much distance between us as possible. (Avoidance)
4. Despite what she did, I would be working hard to bury the hatchet and move forward with our relationship. (Benevolence)
5. I would want to make her pay. (Revenge)
6. I would be striving to achieve reconciliation. (Benevolence)
7. I would be avoiding her. (Avoidance)
8. I would want her to get what she deserves. (Revenge)
9. I would never be able to forgive her. (Eventual: R)
10. How long would it take you to forgive her (please select the response that aligns closest with your thoughts/opinions)? (Time: R)

Self-Forgiveness

You have recently moved into a new apartment. Your new roommate has lived there for several years. Everything is going well with you two, but you recently got a new job, and the job requires that you have reliable transportation. Given that your roommate works only several blocks from your house, she offers to let you use your car.

One day when your roommate leaves the house to run errands, she notices that her car had been left unlocked. She confronts you about it; you apologize, and say that it won't happen again.

Since then your roommate hadn't been paying attention as to whether you had or had not been making sure to lock the car at night, but, on the news, she hears that there has been a rise in car burglaries in your neighborhood. Just to be cautious, she informs you and once again reminds you to be vigilant about locking the car.

The very next morning you awake and see that your roommate's car has been broken into and stripped of all electronics and valuable parts. You had used the car last night and forgot to lock it.

1. How upset would you be if you did this? (Offense Severity)
2. I recently experienced a similar situation. (Recent Experience)
3. I would be trying to keep as much distance between us as possible. (Avoidance)
4. Despite what I did, I would be working hard to bury the hatchet and move forward with our relationship. (Benevolence)
5. I should have to pay for what I did. (Revenge)
6. I would be striving to achieve reconciliation. (Benevolence)
7. I would be avoiding her. (Avoidance)
8. I should get what I deserve. (Revenge)
9. I would never be able to forgive myself. (Eventual: R)
10. How long would it take you to forgive yourself (please select the response that aligns closest with your thoughts/opinions)? (Time: R)
