# Supplementary material for: Personality correlates of dispositional forgiveness: a direct comparison of interpersonal and self-forgiveness using common transgression scenarios
Source: Front Psychol. 2023 Nov 1;14:1218663. doi: 10.3389/fpsyg.2023.1218663 (PMC10646512; doi:10.3389/fpsyg.2023.1218663)
Supplement: Supplementary file 2 [file Data_Sheet_2.PDF]

Supplement B:
Regression Results with Big Five Factors Only (Excluding Explanatory Style)

Table S1. Results of regression analyses without explanatory style for the four forgiveness measures for the interpersonal and self-forgiveness conditions (*N* = 160)

**Interpersonal**	Avoidance			Revenge			Benevolence			Global		
**Forgiveness** (*n* = 82)	*β*	*t*	*p*	*β*	*t*	*p*	*β*	*t*	*p*	*β*	*t*	*p*
Agreeableness	0.03	0.25	.805	**-0.44**	**-3.65**	**<.001**	0.14	1.08	.282	**0.27**	**2.07**	**.041**
Neuroticism	***0.21***	***1.84***	***.069***	0.05	0.44	.658	-0.15	-1.26	.211	-0.17	-1.54	.128
Conscientiousness	0.07	0.57	.571	-0.01	-0.08	.940	-0.06	-0.50	.619	-0.07	-0.58	.565
Openness	-0.10	-0.79	.430	-0.03	-0.28	.783	-0.15	-1.23	.223	0.07	0.57	.568
Extraversion	-0.07	-0.56	.576	-0.05	-0.50	.621	0.17	1.41	.161	0.01	0.10	.924
	$R^2 = 0.08$			$R^2 = 0.24$			$R^2 = 0.08$			$R^2 = 0.14$		
**Self-Forgiveness**	Avoidance			Revenge			Benevolence			Global		
(*n* = 78)	*β*	*t*	*p*	*β*	*t*	*p*	*β*	*t*	*p*	*β*	*t*	*p*
Agreeableness	-0.12	-1.02	.311	**0.25**	**2.03**	**.046**	0.17	1.34	.184	-0.13	-1.10	.275
Neuroticism	**0.22**	**2.05**	**.044**#	0.19	1.65	.104#	-0.02	-0.17	.870	**-0.33**	**-3.18**	**.002**
Conscientiousness	**-0.27**	**-2.28**	**.026**	-0.05	-0.40	.691	0.09	0.71	.481	**-0.31**	**-2.71**	**.008**
Openness	-0.09	-0.78	.436	0.02	0.14	.887	-0.09	-0.78	.440	0.03	0.319	.759
Extraversion	-0.04	-0.41	.681	0.14	1.23	.222	0.09	0.73	.468	-0.14	-1.29	.200
	$R^2 = 0.20$			$R^2 = 0.10$			$R^2 = 0.07$			$R^2 = 0.24$		

Notes: Bolded values indicate $p < 0.05$, whereas bold italics indicate $0.10 > p > 0.05$.
Although statistical conclusions mostly remained the same without explanatory style in the model, these two cases showed differences. Specifically, without explanatory style, neuroticism significantly predicted avoidance motivations, but its unique predictive power for revenge motivations was no longer even marginally significant.

Table S2. Results of regression analyses without explanatory style for the two subscales (Other and Self) of the Heartland Forgiveness Scale (HFS)

	HFS Other (N = 160)			HFS Self (N = 160)		
Measure	β	t	p	β	t	p
Agreeableness	**0.50**	**6.36**	**< .001**	**0.15**	**2.15**	**.033**
Neuroticism	-0.03	-0.37	.714	**-0.45**	**-7.00**	**< .001**
Conscientiousness	-0.07	-0.89	.376	-0.03	-0.45	.657
Openness	0.07	1.00	.317	**0.15**	**2.33**	**.021**
Extraversion	0.01	0.09	.931	**0.21**	**3.21**	**.002**
	$R^2 = 0.26$			$R^2 = 0.41$		

Notes: Bolded values indicate $p < 0.05$, whereas italics indicate $0.10 > p > 0.05$. Statistical conclusions did not change when explanatory style was excluded from regression models
